# Complete Protection against Influenza Virus H1N1 Strain A/PR/8/34 Challenge in Mice Immunized with Non-Adjuvanted Novirhabdovirus Vaccines

**DOI:** 10.1371/journal.pone.0164245

**Published:** 2016-10-06

**Authors:** Ronan N. Rouxel, Emilie Mérour, Stéphane Biacchesi, Michel Brémont

**Affiliations:** VIM, INRA, Université Paris-Saclay, 78350, Jouy-en-Josas, France; Rockefeller University, UNITED STATES

## Abstract

Novirhabdoviruses like Viral Hemorrhagic Septicemia Virus (VHSV) and Infectious Hematopoietic Necrosis Virus (IHNV) are fish-infecting *Rhabdoviruses* belonging to the *Mononegavirales* order. By reverse genetics, we previously showed that a recombinant VHSV expressing the West Nile Virus (WNV) E glycoprotein could serve as a vaccine platform against WNV. In the current study, we aimed to evaluate the potential of the Novirhabdovirus platform as a vaccine against influenza virus. Recombinant Novirhabdoviruses, rVHSV-HA and rIHNV-HA, expressing at the viral surface the hemagglutinin HA ectodomain were generated and used to immunized mice. We showed that mice immunized with either, rVHSV-HA or rIHNV-HA, elicited a strong neutralizing antibody response against influenza virus. A complete protection was conferred to the immunized mice when challenged with a lethal dose of influenza H1N1 A/PR/8/34 virus. Furthermore we showed that although acting as inert antigen in mice, since naturally inactivated over 20°C, mice immunized with rVHSV-HA or rIHNV-HA in the absence of adjuvant were also completely protected from a lethal challenge. Novirhabdoviruses platform are of particular interest as vaccines for mammals since they are cost effective to produce, relatively easy to generate and very effective to protect immunized animals.

## Introduction

Regarding vaccination strategies against virus, vaccine approaches can be divided into two large groups: (i) Live vaccines, mainly consisting of live attenuated viruses and replicating recombinant virus vectors, and (ii) killed or subunit vaccines such as inactivated viruses, virus-like particles (VLP), DNA vaccines, recombinant proteins or subunit particles. Despite the fact that they usually induce a weaker and shorter immune response compared to live vaccines, killed vaccines are safer to use as they have no potential of reversion or reassortment and no risk for immune-compromised host [1, 2, 3]. However, killed vaccines are usually more expensive to produce as they require multiple purification steps, validation of inactivation process and safety control. They also need large amount of specific antigens to be immunogenic and often require several boosts to induce and to maintain a long-term protective immunity [4, 5, 6]. Good examples of such distinct vaccine approaches are the influenza vaccines, which are available both as influenza inactivated viruses (IIV) and live-attenuated viruses (LAV) [7]. Influenza viruses belong to the *Orthomyxoviridae* family. They are negative-sense single-stranded RNA viruses with a segmented genome. Influenza A virus contains 8 genomic segments, each coding for mainly one or two viral proteins. Among them, hemagglutinin (HA) and neuraminidase (NA) are the two influenza virus glycoproteins implicated in the virus entry and release from the cell. HA and NA proteins are also used to classify the influenza viruses into their different serological subtypes, such as H1N1, H3N2 or H5N1 [8]. According to the World Health Organization, seasonal influenza is responsible each year for about 4 million cases of severe illness and up to 500,000 deaths worldwide (http://www.who.int/mediacentre/factsheets/fs211/en/). Efficient vaccine strategies do exist to prevent influenza infections; however, the segmented nature of the viral genome allowing potential reassortment with highly pathogenic strains and its ability to antigenically drift, confer to influenza virus the potential to rapidly emerge as new epidemic or pandemic strain, requiring the development of new vaccines. To thwart this and in order to provide an effective protection to populations, more effective influenza vaccines, greater production capacity, and faster throughput will be required [9]. Novirhabdoviruses are fish-infecting rhabdoviruses belonging to *Mononegavirales* order. Reverse genetics systems for novirhabdovirus have been developed in the past, allowing the viral genome manipulation and the recovery of viable recombinant viruses. Furthermore, the immunogenic potential of novirhabdovirus as a recombinant vaccine platform has already been demonstrated in mice with a recombinant West Nile Virus antigen [10]. Novirhabdovirus platforms offer numerous advantages: 1) they are easy and fast to construct, 2) they grow to high titer in fish cell lines, 3) they can incorporate a large panel of foreign antigens at their surface (glycoproteins or protein domains) by adding the signal peptide and the transmembrane domain derived from the novirhabdovirus glycoprotein to the antigen of interest 4) they are self-adjuvanted and 5) they are naturally inactivated over 20°C and thus safe to use in mammals [11, 12]. In the current study, we aimed to evaluate the potential of the novirhabdovirus platforms as recombinant vaccines against influenza infection in a mouse model.

## Materials and Methods

### Ethics statements

All animal studies were carried out in strict accordance with the European guidelines and recommendations on animal experimentation and welfare. All animal experiment procedures were approved by the local ethics committee on animal experimentation: COMité d’ÉTHique pour l’Expérimentation Animale (COMETHEA INRA N°45) and registered under the number N°02238.01.

To minimize animal suffering and distress, immunization, blood sampling and virus challenge were carried out under anesthesia. Anesthesia was performed by intraperitoneal injection of 150 to 200 μl of a mixture containing 50 mg/kg of ketamine and 10 mg/kg of xylazine.

A lethal challenge typically results in severe disease characterized by huddling, ruffled fur, lethargy, anorexia leading to weight loss, and death. Therefore, mice were monitored for weight loss and survival (twice per day) for 14 consecutive days following the criteria described below in the paragraph of influenza challenge in vaccinated mice. Mice that showed typical infection symptoms and weight loss over 20% were humanely euthanized using carbon dioxide chamber or anesthesia followed by cervical dislocation.

### Cells and viruses

*Epithelioma Papulosum Cyprini* (EPC) cells were maintained at 15°C in GMEM/HEPES 25 mM medium supplemented with 2 mM L-glutamine (PAA) and with 10% fetal bovine serum (FBS) (Eurobio). Wild-type rIHNV 32/87, rIHNV-HA, wild-type rVHSV 23/75 and rVHSV-HA were grown on EPC cell monolayer cultures at 15°C in complete media supplemented with 2% FBS. Virus titers were determined by plaque assay on EPC cells grown in 6-well plates maintained under a 0.35% agarose overlay in duplicate. At 5 to 6 days post-infection, cells were fixed with 10% formol and stained with crystal violet and plaques were counted by eyes. Madin-Darby canine kidney (MDCK) cells (ATCC; CCL-34) were maintained at 37°C in MEM/NaHCO_3_ 25 mM medium supplemented with 2 mM L-glutamine (PAA) and with 5% FBS. Influenza virus H1N1 A/Puerto Rico/8/34 (A/PR/8/34), H1N1 A/WSN/33 and H3N2 A/Udorn/1/2004 were grown on MDCK monolayer cell cultures at 37°C in complete MDCK media supplemented with trypsin TPCK (L-1-tosylamide-2-phenylethyl chloromethyl ketone-treated, Sigma), 2 μg/mL. Influenza titers were determined by plaque assay on MDCK cells grown in 6-well plates maintained under a 0.35% agarose overlay in duplicate. At 4 days post-infection, cells were fixed with 10% formol and stained with crystal violet and plaques were counted by eyes.

### Plasmid constructs and recombinant virus recovery

The recombinant cassette integrated into VHSV was constructed as previously described [10]. For rIHNV-HA, a unique *KpnI* restriction enzyme site was integrated by site-directed mutagenesis (QuikChange Site-directed mutagenesis Kit, Stratagene) using specific primers (**Table 1**) in between nucleoprotein N and polymerase-associated P genes of the full-length genome pIHNV [13]. A portion of pIHNV genome containing the glycoprotein (G) gene and the non-coding regions upstream and downstream of the gene was amplified by PCR and cloned into a pJET1.2/blunt Cloning Vector (Thermo Fisher Scientific). Specific restriction enzyme sites were integrated by site-directed mutagenesis in the pJET-G at the beginning and the end of the signal peptide (SP) and at the beginning and the end of the transmembrane domain (TM). *KpnI* restriction enzyme sites were also integrated in the middle of the non-coding region on each side of the G gene and the final construct was verified by nucleotide sequencing. G gene was modified by site-directed mutagenesis using QuikChange® Site-Directed Mutagenesis Kit (Stratagene) to introduce *NheI* and *Pfl23II* restriction sites at the end of the signal peptide and at the beginning of the transmembrane domain sequences, respectively. The expression cassette was then inserted into the full-length genome using the *KpnI* restriction enzyme site. The portion of the influenza hemagglutinin gene encoding the extracellular domain of the glycoprotein (1545 nucleotides / 515 amino acids in length GenBank: CY009444.1, [14]) was amplified by reverse transcription-polymerase chain reaction (RT-PCR) from the genomic viral RNA extracted from the A/PR/8/34 H1N1 strain using the QIAamp Viral RNA Mini Kit (Qiagen) and specifics primers (**Table 1**). Amplified HA PCR product was cloned into pJET 1/2 plasmid and sequenced to check the integrity of the nucleotide sequence prior to the insertion into the pIHNV or pVHSV cassettes. The rVHSV-HA and rIHNV-HA were readily recovered by transfection of pVHSV-HA or pIHNV-HA together with the expression plasmids pT7-N, pT7-P and pT7-L derived from VHSV or IHNV, respectively, in EPC cells infected with vTF7-3 vaccinia virus [15], as previously described (for a review see [16]). Viral titers were determined after 2 passages on EPC cells.

**Table 1:** Primers used in the study. Restriction enzyme sites generated by site-directed mutagenesis or by PCR are boldfaced and underlined.

Primer name	Sequence 5’ to 3’	Restriction enzyme site
Mut IHNV-KpnI	GACCCATCCAGATGTTTAC**GGTACC**CTGACTCTTAGATAGAAAAAAATG	KpnI
IHNV-G-SP-NheI-F	CTGATCACCTGCGGAGCA**GCTAGC**CAAACCGCCAAACCCG	NheI
IHNV-TM-Pfl23II-R	ATTCGACCTTCAATCA**CGTACG**TGGAGTTTCTGGCCCACAA	Pfl23II
VHSV-TM-Eco72I-R	CCCACAACCCCCATCCCAGA**CACGTG**ACCGCTGAACAAAACTCC	Eco72I
HA-SP-NheI-F	AGAAATCGT**GCTAGC**HACACAATATGTATAGGCTACC	NheI
HA-TM-Eco72I-R	TCTATTACG**CACGTG**AATCTGATAGATCCCCATTGATTCC	Eco72I
HA-TM-Pfl23II-R	TCTATTACG**CGTACG**AATCTGATAGATCCCCATTGATTCC	Pfl23II
GS-G-IHNV F2	ATAAATCGT**GGTACC**CTGACTCTTAGATAGAAAAAAATGGCACTATAGT	KpnI
GS-G-IHNV F1	GGCACTATAGTGCTTTTCAACT**ACTAGT**ATGGACACCACGATCACC	SpeI
GE-G-IHNV R2	ATTACG**GGTACC**GTAAACATCTGGATGGGTCGAGGGGCGGGAGAGGAAAAAGGAGGGGG	KpnI
GE-G-IHNV R1	GAGGAAAAAGGAGGGCGGGGCTGTTTGTGT**TACGTA**TTAGGACCGGTTT	SnaBI

### Indirect immunofluorescence analysis on fixed and living cells

EPC cells grown in 24-well plates were infected with the novirhabdoviruses-HA (passage 2, MOI of 2). At 24h post-infection, cells were fixed with a mixture of ethanol and acetone (1:1, vv) at -20°C for 20 min and washed with PBS. Primary mouse monoclonal antibodies H36-26 against HA1 from A/PR/8/34 influenza virus strain kindly provided by Dr Jonathan W Yewdell [17] was incubated in PBS-Tween 0.05% for 45 min at room temperature (RT) and washed 3 times with PBS-Tween 0.05%. Cells were then incubated with Alexa Fluor 488-conjugated anti-mouse immunoglobulins diluted to 1:3,000 (Invitrogen) in PBS-Tween 0.05% for 45 min at RT. Cell monolayers were then visualized with a UV-light microscope (Carl Zeiss).

For live cells, infected cell monolayers were directly incubated with primary mouse antibodies in GMEM 10% FBS culture medium for 45 min at RT. After 3 washes with the same medium, cells were incubated with Alexa Fluor 488-conjugated anti-mouse immunoglobulins (dilution 1:3,000) for 45 min at RT. Three washes were performed and cell monolayers were then visualized with a UV-light microscope (Carl Zeiss).

### Virus production and purification

Wild-type and recombinant novirhabdoviruses were mass produced in EPC cells, clarified by low-speed centrifugation (4,000 rpm for 15 min), concentrated 10 fold by ultracentrifugation at 24,000 rpm in a SW28 Beckman rotor for 90 minutes and finally purified by ultracentrifugation at 34,000 rpm in a SW41 Beckman rotor for 4 hours through a 25% (w/v) sucrose cushion in TEN buffer (10 mM Tris-HCl [pH = 7.5], 150 mM NaCl, 1 mM EDTA [pH = 8]). Influenza virus were mass produced in MDCK cells, inactivated by UV-illumination at 254 nm for 5 minutes (UVitec) and then purified by ultracentrifugation at 34,000 rpm in a SW41 Beckman rotor for 4 hours through a 25% (w/v) sucrose cushion. Virus pellets were resuspended in TEN buffer and the viral protein yield of each preparation was quantified by using the Micro BCA assay protein quantification Kit (Pierce) in accordance with the manufacturer’s instructions.

### SDS-polyacrylamide gel electrophoresis and Western blot assay

Aliquots of sucrose-purified recombinant viruses or UV-inactivated sucrose-purified influenza virus were separated on a sodium dodecyl sulfate 12% polyacrylamide gel (SDS-PAGE; Life technologies) and electrotransferred onto a polyvinylidene difluoride membrane (Immobilon-P; Millipore) using a semi-dry electroblotting system (Biorad). The membrane was saturated in Tris-Buffer Saline containing 0.05% of Tween 20 (TBST) supplemented with 5% of skim-milk for 1h at room temperature (RT), then incubated with antibodies from mouse hybridoma supernatant directed against HA [17] in TBST 5% milk (dilution 1:10) for 1h at RT. After three washes with TBST, the membrane was incubated for 1h at RT with horseradish peroxidase-conjugated anti-mouse antibody (1:10,000; P.A.R.I.S.) in TBST. After extensive washing with TBST, peroxidase activity was revealed by incubation with ECL Western Blotting Detection Reagents (ECL; Pierce) according to the manufacturer’s instructions.

### Serum neutralization test (SNT)

Neutralizing antibodies (NAB) were quantified by serum neutralization test. Serial dilutions of heat-inactivated (30 min at 56°C) serum samples starting at a dilution of 1:10 were incubated with 100 TCID_50_ of influenza virus and incubated for 5 min before adding on MDCK cell monolayers. All the seroneutralization assays were performed in duplicate. At 3 days post-infection, cells were fixed with 10% formol and stained with crystal violet. NAB titer are expressed as the reciprocal of the highest dilution of serum at which cytopathic effect (CPE) on infected cell could be observed.

### Mice immunization

For the first animal experiment (experiment #1), groups of 6 week-old female BALB/c mice (n = 8) (Janvier Labs) were immunized subcutaneously in the back neck with 30 μg of purified rIHNV-HA, rVHSV-HA or rVHSV-wt resuspended in 100 μL of TEN buffer supplemented with Freund’s adjuvant (complete form for the first immunization and incomplete form thereafter) at a 1:2 ratio [10]. In this first animal experiment, two mice died before the first immunization during the pre-immunization blood sampling at day -3 because of human mistake. For the second animal experiment (experiment #2), groups of 6 week-old female BALB/c mice (n = 8) were immunized intramuscularly in the right quadriceps with 30 μg of purified rIHNV-HA, rVHSV-HA, rVHSV-wt or rIHNV-wt resuspended in 100 μl of TEN buffer only. For both animal experiments, mice were immunized three time at two weeks interval with three times the same recombinant viruses or an alternation of the two recombinant viruses rIHNV-HA and rVHSV-HA. Before, during and after immunization, blood samples were collected from the sub-mandibular vein. All the injected animals were daily examined during the time of immunization. No signs of any pain or toxicity were observed.

### Influenza challenge in vaccinated mice

Each group of 6 week-old female BALB/c mice was infected intranasally with 5x10^4^ PFU of H1N1 A/PR/8/34 hen’s egg influenza stock, (gift from Dr Nicolas Bertho). Animals were monitored daily for signs of disease. To assess clinical disease severity, a four-step grading system was used. For posture, 0 represented normal, 1 slow movement, 2 back slightly curved and fur slightly tousled, 3 back curved and tousled fur. Activity was graded 0 for normal, 1 for calm, 2 for delay to stimuli and 3 for inactive. Weight loss was classified as 0 for 0 to 10% loss, 1 for 10 to 15% loss, 2 for 15 to 20% loss, or 3 for more than 20% loss. Dehydration (skin tenting) was classified as 0 for none, 1 for weak, 2 for moderate and 3 for severe. Ringer’s solution was injected subcutaneously after weighing when mice were dehydrated.

## Results

### Recovery of recombinant rIHNV-HA and rVHSV-HA

**Fig 1:**
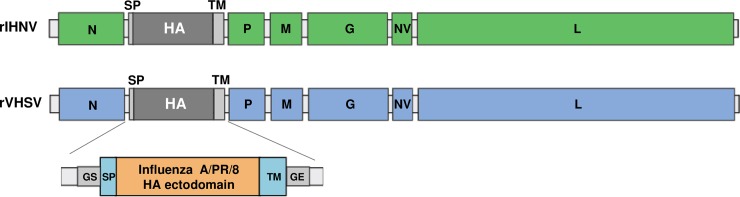
Schematic representation of the engineered rIHNV-HA and rVHSV-HA genomes. GS: gene start; GE: gene end; SP: signal peptide; TM: transmembrane.

Recombinant rIHNV-HA and rVHSV-HA were generated as described previously [10]. Briefly, *KpnI* unique restriction enzyme sites were introduced by site-directed mutagenesis in the non-coding regions between N and P genes. Using this unique restriction enzyme site, an expression cassette was added to the full-length genome following several cloning steps. The expression cassettes contain successively the signal peptide (SP) sequence derived from the IHNV glycoprotein G gene, the coding sequence for the ectodomain and stalk from the influenza H1N1 A/PR/8/34 hemagglutinin (HA) and the transmembrane sequence (TM) from either IHNV or VHSV G gene. The expression cassettes were flanked with the gene start and gene end signals of IHNV or VHSV in order to be recognized by the viral polymerase and to direct the efficient expression of heterologous genes (Fig 1). All recombinant viruses were readily recovered using the established reverse genetics systems for VHSV and IHNV as previously described (for a review see [16]). Both recombinant viruses were amplified through 2 passages in fish EPC cells. Titers reached 2 x 10^8^ PFU/mL for rVHSV-HA and 2 x 10^7^ PFU/mL for rIHNV-HA, which are about 10-fold less than the wild-type viruses: 10^9^ PFU/mL for wtVHSV and 2 x 10^8^ PFU/mL for wtIHNV.

### Both recombinant viruses express influenza HA antigen in fish cells

**Fig 2:**
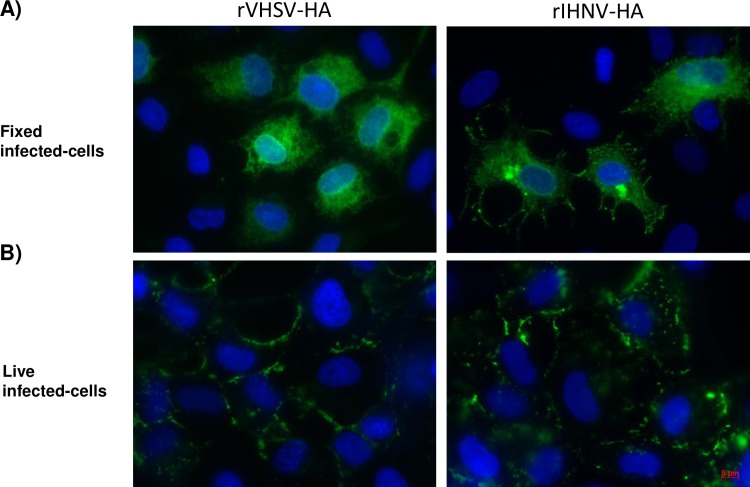
Expression of HA antigens in rVHSV-HA or rIHNV-HA infected cells. The expression of HA antigens was assessed by indirect immunofluorescence assays on EPC cells. The cells were infected with either rVHSV-HA or rIHNV-HA and incubated at 14°C. (A) At 24 h post-infection, cells were fixed and permeabilized with alcohol/acetone and HA expression was detected using a monoclonal antibody against HA1 (H36-26). (B) At 48 h post-infection, membrane expression of HA antigen was visualized on live cells in PBS using H36-26 monoclonal antibody (magnification x40).

To assess the correct expression of recombinant HA antigen by recombinant IHNV-HA and VHSV-HA viruses, EPC cells were infected with each recombinant Novirhabdovirus at a multiplicity of infection (MOI) of 0.1 and 0.01, respectively. At 24 h or 48 h post-infection, the level of expression of the recombinant HA protein was evaluated by indirect immunofluorescence on fixed or live infected cells (Fig 2). As shown in Fig 2A, both recombinant viruses expressed the HA protein in the cytoplasm of infected EPC cells. As Novirhabdoviruses bud from the cell membrane, we analyzed the expression of HA at the surface of live infected EPC cells to ensure the correct addressing of the antigen (Fig 2B). Both rIHNV-HA and rVHSV-HA viruses expressed and correctly addressed HA at the surface of infected EPC cells.

### Both recombinant viruses present the recombinant influenza HA antigen on their surface

**Fig 3:**
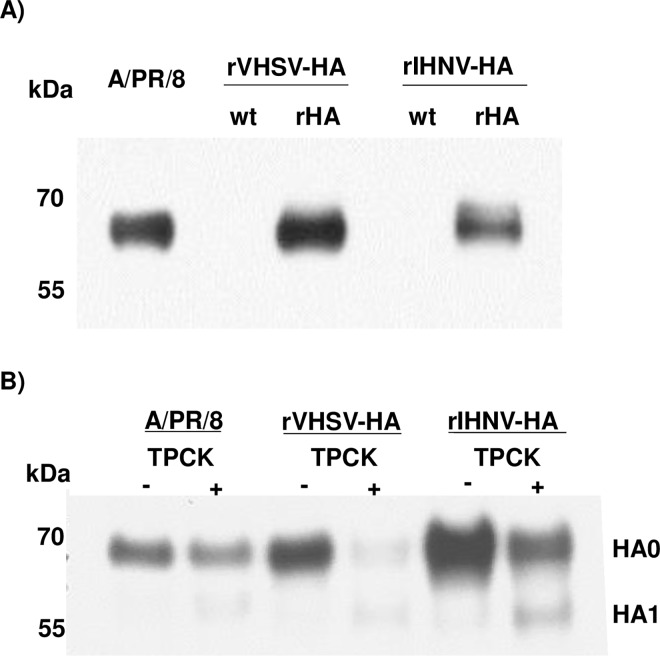
Analysis of HA antigens incorporation in recombinant virus particles. Proteins of sucrose gradient-purified viral particles were separated on a 12% polyacrylamide gel. A) Six micrograms of total viral proteins were denaturated, loaded and migrated on SDS page gel. The gel was electrotransfered onto a PVDF membrane and HA antigens were detected with monoclonal antibody directed against HA. B) Three to six mg of total viral proteins were incubated at 37°C with or without 2 mg/mL of Trypsin-TPCK for 5 min prior denaturation, loading and migration on SDS-PAGE as in A.

The incorporation efficiency of foreign antigens at the surface of recombinant Novirhabdoviruses with an expression cassette between N and P genes has been previously demonstrated [10]. The HA antigen could not be clearly visualized on SDS-PAGE after Coomassie blue staining as it co-migrated with the G glycoproteins of IHNV and VHSV platforms (data not shown). We therefore validated the expression of the HA antigen at the surface of the two novirhabdovirus-HA platforms by Western-blot assay and by immunogold staining on purified recombinant viruses. The Fig 3A shows that rIHNV-HA and rVHSV-HA both express the HA antigen at the expected size of 62 kDa. When recombinant viruses were incubated for 5 min in presence of Trypsin-TPCK, HA0 antigen were cleaved and HA1 subunit could be detected by Western-blot assay similarly to influenza A/PR/8/34 virus, suggesting a folding of the recombinant HA such as the trypsin cleavage site remained accessible (Fig 3B).

**Fig 4:**
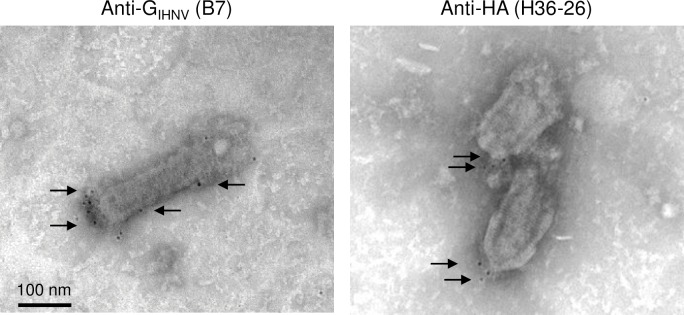
Detection of HA antigen at the surface rIHNV-HA particle through immunogold staining. Sucrose gradient-purified viral particles were adsorbed on electron microscopy nickel grids. After fixation, the glycoprotein G of IHNV and HA antigen were detected using specific mouse monoclonal antibodies and anti-mouse secondary antibody coupled with gold particles (black dots of 5 nm in diameter). After negative staining, recombinant viral particles were observed by transmission electron microscopy. Magnification x40 000.

As previously shown for rVHSV expressing WNV-E derived antigen [10], visualization of recombinant HA antigen at the surface of the virus particle could be achieved by immunogold staining for both rVHSV-HA (data not shown) and rIHNV-HA (Fig 4).

### Recombinant novirhabdoviruses express large amount of recombinant HA antigens

**Fig 5:**
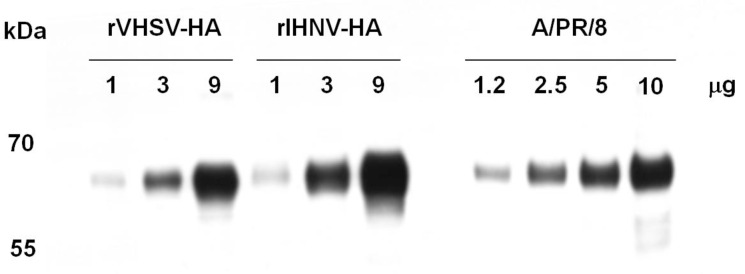
Estimation of the amount of recombinant HA antigens expressed on recombinant viruses. Proteins of sucrose gradient-purified viral particles were separated on a 12% polyacrylamide gel. Serial amount of total viral proteins were loaded and migrated on SDS-PAGE. Proteins concentration was estimated using Micro BCA assay protein quantification Kit. The gel was electrotransfered onto a PVDF membrane and HA antigens were detected with a monoclonal antibody directed against HA.

We next evaluated the amount of HA antigen comparatively to that of an UV-inactivated purified influenza A/PR/8/34 parental strain virus. Serial dilutions of purified rVHSV-HA, rIHNV-HA (both produced in EPC cells) and influenza A/PR/8/34 viruses (produced in MDCK) were analyzed on SDS-PAGE. Following a Western blot assay using an anti-HA mAb (H36-26), the amount of HA antigens inserted in the recombinant virus envelopes was evaluated using the image processing program ImageJ (National Institute of Health, USA; http://rsb.info.nih.gov/ij/) (Fig 5). The quantities of recombinant HA antigens in recombinant novirhabdoviruses were similar to whole influenza A/PR/8/34 purified virus produced in MDCK cells and based on a BCA-estimated viral protein concentration.

### Adjuvanted recombinant novirhabdovirus-HA platforms induce a neutralizing immune response against HA A/PR/8/34 in BALB/c mice

**Fig 6:**
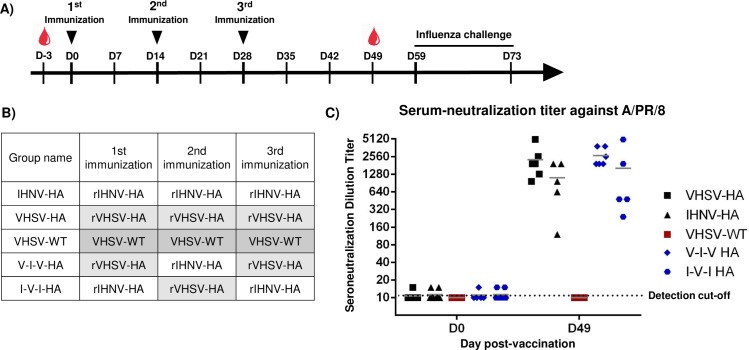
Recombinant novirhabdoviruses induce a neutralizing immune response against HA A/PR/8/34 in BALB/c mice. A) Schedule of immunization and B) Groups of BALB/c mice (n = 8) six-week old were immunized subcutaneously, 3 times at two-week interval with 30 μg of purified recombinant novirhabdoviruses. Mice sera were taken at day -3 and day 49. At day 59, mice were challenged with a lethal dose of 5x10^4^ PFU of influenza A/PR/8/34 virus. Course of the infection was monitored daily for two weeks. C) Seroneutralization antibody assay against influenza HA A/PR/8/34. Heat-inactivated sera of immunized mice at day 49 were used to evaluate the amount of serum neutralization antibodies against the influenza virus. Titers are expressed as reciprocals of the highest antibody dilution at which cytopathic effects appeared. Bars represent the mean titer of 8 animals in each group.

Six-weeks old BALB/c mice were immunized subcutaneously with three doses, at two-week intervals, with purified recombinant novirhabdovirus platforms with adjuvant (Experiment #1) following the protocols depicted in Fig 6. Mice were randomly divided into groups of 8 as shown in Fig 6B (except for 2 groups with 7 mice as explained in materials and methods). Two groups were immunized with either rIHNV-HA or rVHSV-HA and two groups were immunized with rIHNV-HA and rVHSV-HA in alternation. The rational for the latest groups was to evaluate the impact of alterning two antigenicaly distinct platforms but expressing the same antigen of interest (see discusion). Control group was immunized with an empty recombinant platform rVHSV-wt. During the immunization, none of the mice showed any side effects and all mice had normal behavior. To evaluate the humoral immune response induced by novirhabdovirus-HA platforms, blood samples were collected after two weeks following the last boost and serum neutralizing antibody (NAB) titer against A/PR/8/34 virus was assayed. All mice immunized with recombinant novirhabdovirus-HA platforms mounted a strong antibody response with significant neutralizing antibody titers directed against HA influenza virus A/PR/8/34. As shown in Fig 6C, mice vaccinated subcutaneously with adjuvanted recombinant platforms presenting HA antigens mounted a mean neutralizing antibody response from 1:1112±356 (arithmetic mean titer +/- standard error) to 1:2667±385 at day 49 with no significant differences between groups based on two-way ANOVA test with Tukey’s multiple comparison tests. Mice immunized with the empty recombinant platforms had neutralizing antibody below our detection limits (1:10). Finally, the use of three doses of the same platform did not affect the NAB titers compared to alternance of antigenicaly distinct platforms bearing the same recombinant antigen (Fig 6C).

In order to assess the level of heterosubtypic protection induced by immunization with the recombinant novirhabdovirus-HA platforms, we performed NAB assays against another H1N1 influenza virus (A/WSN/33) or a H3N2 influenza virus (A/Udorn/1/2004). Equivalent amounts of serum were pooled together in a single mix to evaluate immune response of the whole group. None of the groups showed any detectable neutralizing immune response against heterosubtypic influenza viruses (detection limit 1:10), highlighting the specificity of the immune response induce by recombinant novirhabdovirus-HA (data not shown).

### Mice immunized with adjuvanted recombinant novirhabdovirus-HA are fully protected against an A/PR/8/34 influenza challenge

**Fig 7:**
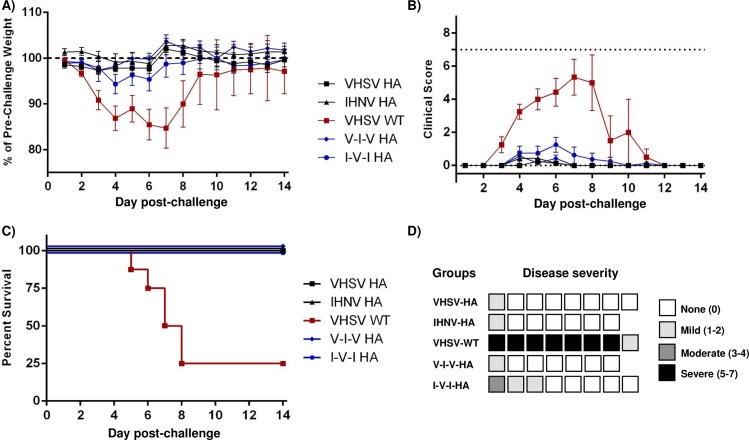
Lethal challenge experiments. Mice previously vaccinated were challenged intranasally with a lethal dose of 5x10^4^ PFU of influenza A/PR/8/34 virus. A) Weight change and B) Clinical score over the course of the disease. Mice were weighted and followed daily for two weeks. In graphs A and B are presented the mean values of each group with standard error. C) Survival curves of mice immunized with rVHSV-HA and rIHNV-HA and challenged with influenza. D) Disease severity. Each box represents one mouse and the severity of its disease when clinical signs were detected for more than 24h. Black indicates severe (maximum clinical score from 5 to 7), dark gray: moderate (3–4), light gray: mild (1–2) and white indicates that the mouse did not show any clinical sign for more than 24h.

To assess the protection conferred by vaccination with recombinant novirhabdovirus-HA, immunized mice were challenged intranasally with a lethal dose of 5x10^4^ PFU of influenza virus H1N1 A/PR/8/34. Course of the infection was followed for 14 days with daily monitoring of weight loss and clinical scores (Fig 7). All mice immunized with recombinant novirhabdovirus-HA survived the infection and showed no or only minor weight loss and no or only limited clinical signs of disease. In contrast, mice immunized with empty recombinant platform (rVHSV-wt) had severe weight loss and showed severe signs of illness. These animals had to be euthanized for ethical reasons between 4 to 8 days post-infection, with the exception of two mice which recovered despite noticeable illness (Fig 7B). In this experiment, only 6 out of 28 vaccinated mice showed signs of illness for more than 24h ranging from mild (5/30, mainly dehydration) to moderate (1/30) (Fig 7D).

### Non-adjuvanted recombinant novirhabdovirus-HA platforms induce a neutralizing immune response against HA A/PR/8/34 in BALB/c mice

**Fig 8:**
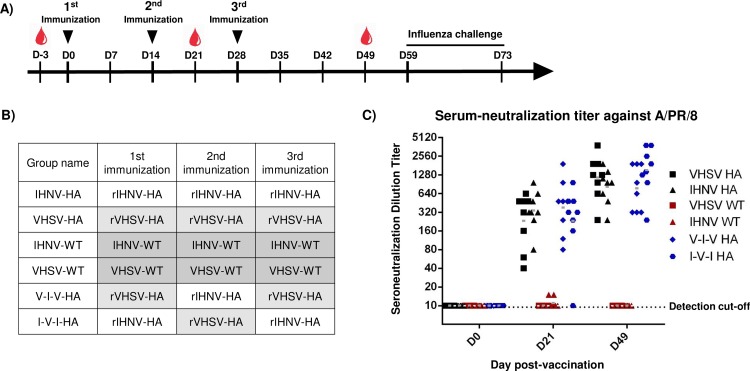
Non-adjuvanted recombinant novirhabdoviruses induce a neutralizing immune response against HA A/PR/8/34 in BALB/c mice. A) Schedule of immunization and B) groups of BALB/c mice (n = 8) six-week old were immunized subcutaneously with 30 μg of purified recombinant novirhabdoviruses three times at two week interval. Mice sera were taken at day -3, day 21 and day 49. At day 59, mice were challenged with a lethal dose of 5x10^4^ PFU of influenza A/PR/8/34 virus. Course of the infection was monitor daily for two weeks. C) Seroneutralization antibody assay against influenza HA A/PR/8/34. Heat-inactivated sera of immunized mice at day 49 were used to evaluate the amount of serum neutralization antibodies against influenza virus. Titers are expressed as reciprocals of the highest antibody dilution at which cytopathic effects appeared. Bars represent the mean titer of 8 animals in each group.

In a second experiment, six-weeks old BALB/c mice were immunized intramuscularly with three doses, at two-week intervals, with purified recombinant novirhabdovirus platforms without adjuvant (Experiment #2). As in Experiment #1 mice were randomly divided into groups of 8 as shown in Fig 8B. Two groups were immunized with either rIHNV-HA or rVHSV-HA and two groups were immunized with rIHNV-HA and rVHSV-HA in alternation. Mice vaccinated with non-adjuvanted recombinant platforms had a mean NAB titer from 1:333±79 to 1:595±213 after two doses at day 21 and a mean titer of 1:970±190 to 1:2010±465 after three doses at day 49 (Fig 8C).

### Mice immunized with non-adjuvanted recombinant novirhabdovirus-HA are fully protected against an A/PR/8/34 influenza challenge

**Fig 9:**
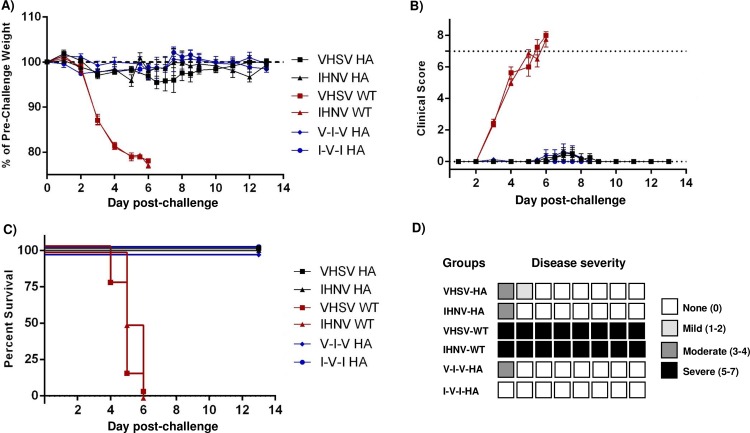
Lethal challenge experiments. Mice previously vaccinated with non-adjuvanted recombinant viruses were challenged intranasally with a lethal dose of 5x10^4^ PFU of influenza A/PR/8/34 virus. A) Weight change and B) Clinical score over the course of the disease. Mice were weighted and followed daily for two weeks. In graphs A and B are presented the mean values of each group with standard error. C) Survival curves of mice immunized with rVHSV-HA and rIHNV-HA following challenge. D) Disease severity. Each box represents one mouse and the severity of its disease when clinical signs were detected for more than 24h. Black indicates severe (maximum clinical score from 5 to 7), dark gray: moderate (3–4), light gray: mild (1–2) and white indicates that the mouse did not show any clinical sign for more than 24h.

As for the experiment #1, immunized mice were challenged intranasally with a lethal dose of 5x10^4^ PFU of influenza virus H1N1 A/PR/8/34. Course of the infection was followed for 14 days with daily monitoring of weight loss and clinical scores (Fig 9). All mice immunized with recombinant novirhabdovirus-HA survived the infection and showed no or only minor weight loss and no or only limited clinical signs of disease. In contrast, mice immunized with empty recombinant platforms (rVHSV-wt or rIHNV-wt) had severe weight loss and showed severe signs of illness. These animals had to be euthanized for ethical reasons between 4 to 8 days post-infection, For experiment #2, only 4 out of 32 vaccinated mice showed signs of disease for more than 24 h ranging from mild (1/32) to moderate (3/32) (Fig 9D).

Globally, for both experiments, an average of only 16% of vaccinated mice showed sign of disease for more than a day, mainly transient dehydration or calm activity. In both animal experiments, all vaccinated mice were completely protected against a lethal influenza infection.

## Discussion

In the current study, we aimed to generate recombinant novirhabdoviruses expressing the hemagglutinin ectodomain of influenza virus, in order to characterize their immunogenicity and the protection they induced in a mouse model. For that, genomes of VHSV and IHNV have been engineered such as an additional cistron has been inserted between the N and P genes. This cistron encodes the influenza HA-derived ectodomain fused to the signal peptide and the transmembrane region derived from the VHSV and the IHNV G glycoproteins. Respective recombinant viruses, named rVHSV-HA and rIHNV-HA, were successfully recovered through the available reverse genetics systems. Insertion of approximately 1700 nucleotides in between N and P genes of VHSV or IHNV genome did not affect the fitness of the viruses. Recombinant viruses displayed the same morphology as wild-type viruses and were produced at similar titers as those previously observed for rVHSV bearing WNV E-derived antigen, approximately a log lower compared to rVHSV-WT [10]. The genetic stability of the recombinant virus genomes was confirmed by sequencing analysis after four passages in fish cells (data not shown). We next demonstrated the efficient expression and incorporation of HA at the surface of the virus particle and their immunogenic and protective potential in mice. Whether the mice were immunized subcutaneously with Freund’s adjuvant or intramuscularly without any adjuvant, all mice vaccinated on a three dose schedule were fully protected against a lethal challenge with the parental strain of influenza H1N1. Neutralizing antibody titers measured at day 21 (experiment #2 without adjuvant), after the second boost of vaccination ranged from 1:40 to 1:1280, suggesting that two boosts might be sufficient to induce a complete protection (Fig 8C).

Adjuvants are compounds added to the vaccine dose to improve its efficiency by stimulating the immune response at the site of injection [18]. Recently, except for the common local reactions, adjuvants have been unwarrantedly suspected to induce severe complications restraining their acceptability in the community [18, 19, 20]. Moreover the use of adjuvant represents an additional cost of clinical testing and production which makes vaccines that do not require adjuvant of particular economic interest [18]. It is known that influenza vaccines have a higher immunogenicity in the population when adjuvanted subunits virions are used but they are usually more reactogenic than non-adjuvanted whole virion vaccines [7, 21, 22]. Geeraedts et *al*. [23] have shown that in unprimed individuals, inactivated whole viruses are more immunogenic than subunits H5N1 vaccines, due to the presence of single-stranded RNA within the virion and its recognition by TLR7 which triggers the immune response. In the present study we showed that adding of adjuvant seems useless by intramuscularly injection of purified whole-virus VHSV-HA or IHNV-HA, since immunization without any additive was sufficient to induce a strong protective immune response.

Although killed or subunit vaccines are usually less immunogenic than live-attenuated vaccines, they can however induce a strong targeted immune response even in immuno-compromised individuals. Several studies suggest that an exact match between vaccine strain and pandemic strain might not be necessary to contain an influenza pandemia and it has been estimated that an influenza pandemic vaccine with as low as 30% efficacy could have an impact on limiting mortality and morbidity [24]. Thus the use of an engineered HA antigen antigenically optimized obtained by reverse genetics could lead, in an emergency state, to a first vaccination step and could be sufficient to limit the burden of the disease. Also the use of recombinant inactive platform is of particular interest in prime-boost strategies. The use first of an inactive vaccine to induce a first level of protection, followed by a second immunisation with an attenuated virus to induce a wider immune response could prevent the risks commonly associated with live-attenuated vaccines [25, 26]. It is known that highly conserved epitopes, such as in the HA stalk, can elicit a cross-protective immunity [27]. We can therefore make an assumption that a first vaccination with a novirhabdovirus-HA^stalk^ platform followed by vaccination with a LAV vaccine would advantageously optimize the immune response towards highly conserved antigenic target within the stalk of HA. Based on the immunogenic original antigenic sin hypothesis, highly conserved antigenic targets should be favored, while inducing during the second immunization an immune response against other influenza antigens [25, 28, 29]. In our study, we did not detect higher neutralizing antibody titers when alternative antigenically distinct platforms were used to present the same antigen (Figs 6C and 8C). In both cases, after immunization with the same or an alternation of both platforms, mice mounted a similar humoral immune response. The benefits of an alternation of immunogenic platforms could be investigated more in detail with longer time delays in between boost.

The present study validates for the first time that, as for rVHSV, rIHNV is also an efficient antigen-presenting platform allowing efficient expression of a recombinant antigen within the virus particle. Both rVHSV and rIHNV are interesting vaccine platforms, inducing upon a three doses immunization a total protection against a lethal dose of influenza virus in all vaccinated animals. There are several advantages to use novirhabdoviruses as recombinant platforms, in terms of safety, cost and time-saving. Regarding safety issues, VHSV and IHNV are unable to replicate over 20°C [11, 12, 30], therefore they do not need inactivation procedure and thus prevents possible alteration of the antigen [31, 32], and makes them safe to use in warmblood animals. Preliminary results suggest a correct folding of the antigen at the surface of the virus particle but deeper studies will be necessary to validate this point especially if conformational epitopes are targeted. Moreover, novirhabdoviruses grow at high titer and large amount of antigen material can be obtained which is of particular interest for mass-production. Finally, once the nucleotide sequence of the targeted antigen is known, recombinant virus stocks can be produced in routine in few weeks. The use of standardized expression cassettes is of particular interest to fasten the recovery of immunogenic platforms in face of an emerging disease.

In conclusion, multipling the alternatives, the expression platforms and the production systems are crucial to ensure a fast and adapted vaccine production response in face of a pandemic, not only in regards of influenza. The use of novirhabdovirus platforms which can be mass produced in fish cell lines represents an attractive, efficient and safe alternative for inactivated vaccine production. Moreover, the Novirhabdovirus platforms may be developed as vaccines against a large panel of virus including viruses not adapted to tissue-culture or difficult to grow in mass.
